# Complete Genome Sequence of Plant Growth-Promoting Bacillus pumilus TUAT1

**DOI:** 10.1128/MRA.00076-19

**Published:** 2019-05-23

**Authors:** Shin Okazaki, Naoto Sano, Tetsuya Yamada, Kazuo Ishii, Katsuhiro Kojima, Salem Djedidi, María D. Artigas Ramírez, Kun Yuan, Motoki Kanekatsu, Naoko Ohkama-Ohtsu, Yuu Hirose, Kenshiro Oshima, Masahira Hattori, Tadashi Yokoyama

**Affiliations:** a Institute of Agriculture, Tokyo University of Agriculture and Technology, Tokyo, Japan; b Graduate School of Agriculture, Tokyo University of Agriculture and Technology, Tokyo, Japan; c Faculty of Agriculture, Tokyo University of Agriculture and Technology, Fuchu, Tokyo, Japan; d Institute of Global Innovation Research, Tokyo University of Agriculture and Technology, Fuchu, Tokyo, Japan; e Department of Environmental and Life Sciences, Toyohashi University of Technology, Aichi, Japan; f Center of Omics and Bioinformatics, Graduate School of Frontier Sciences, University of Tokyo, Tokyo, Japan; University of Maryland School of Medicine

## Abstract

Bacillus pumilus TUAT1 was isolated from soil in a university research field. Strain TUAT1 has the ability to promote the growth of plants, including that of rice, and has been commercialized as a biofertilizer. Here, we sequenced and annotated the genome of TUAT1 to understand the molecular mechanisms underlying its plant growth promotion.

## Abstract (precis)

Bacillus pumilus TUAT1 was isolated from soil in a university research field. Strain TUAT1 has the ability to promote the growth of plants, including that of rice, and has been commercialized as a biofertilizer.

## ANNOUNCEMENT

We screened plant growth-promoting strains from field soils at the Tokyo University of Agriculture and Technology (Tokyo, Japan) and isolated a strain, TUAT1, which was identified as Bacillus pumilus by 16S rRNA gene analysis. TUAT1 promotes the growth of several plants, including that of rice (Oryza sativa L.) and Brassica species (1–5). An inoculant was developed using TUAT1 that has been commercialized in Japan since 2017. So far, the growth-promoting traits of TUAT1 have not been elucidated. Here, we performed whole-genome sequencing of TUAT1 to understand the molecular mechanisms responsible for the strain’s plant growth-promoting ability and effective use as a biofertilizer.

TUAT1 was grown on LB medium (6) at 37°C overnight. The cell pellets were lysed in sodium dodecyl sulfate (final concentration, 1.5%) and proteinase K (final concentration, 1 mg/ml) at 50°C for 1 h, followed by extraction with cetyl trimethyl ammonium bromide (CTAB)-NaCl as described by Wilson (7). Whole-genome sequencing was performed using a 454FLX+ instrument (Roche). A shotgun library and an 8-kb paired-end library were prepared using a GS FLX+ library preparation kit (Roche) and GS FLX paired-end kit (Roche), respectively. These libraries were sequenced on the GS FLX+ instrument, yielding 566,529 shotgun reads (259 Mb) and 188,461 paired-end reads (71 Mb). GS FLX data processing was performed using the Roche GS FLX software (version 2.7). Base-called reads were trimmed and filtered for quality. These reads were assembled using Newbler version 2.8 (Roche) with default parameters, resulting in two scaffolds of 3.7 Mb and 3.0 kbp containing 18 contigs (>500 bp) with an *N_5_*_0_ contig size of 499 kbp. The assembled data were further polished using sequencing data produced by Gene Analyzer IIx (Illumina, CA), namely, 51,850,857 reads of 102 bp. The remaining 21 gaps were closed by Sanger sequencing.

The length of the whole genome obtained was 3,723,433 bp with a G+C content of 41.4%. Genome annotations were done using the Prokaryotic Genome Annotation Pipeline (PGAP) algorithm of the National Center for Biotechnology Information (NCBI) (8) using default parameters. The annotated genome contained 3,888 genes, with 3,778 total coding sequences (CDS), 3,732 protein-coding genes, 46 pseudogenes, 81 tRNA genes, 24 rRNA genes, and 5 noncoding RNA genes. The whole-genome structure and 16S rRNA gene sequence of TUAT1 were most similar to those of *B. pumilus* MTCC B6033 (3,763,493 bp, 3,659 genes, 81 tRNAs, 6 rRNA clusters, and 41.4% G+C content), which was originally isolated from soil in India as a biocatalyst for the stereospecific oxidation of β-lactams (9).

TUAT1 has been shown to exert plant growth promotion effects on several plants, but the underlying mechanism has not yet been elucidated. Genome analysis of TUAT1 revealed the presence of several genes that have been reported to be involved in the plant growth promotion effects, including those involved in indole-3-acetic acid synthesis (*ysnE*, locus tag BTUAT1_32950, and *yhcX,* BTUAT1_08910), siderophore biosynthesis protein (BTUAT1_10350), or acetoin metabolism (*acuABC*, locus tags BTUAT1_26940, BTUAT1_26950, and BTUAT1_26960). Interestingly, our preliminary experiments indicated that the spore of TUAT1 exhibited a higher plant growth promotion activity than vegetative cells of TUAT1 (10). Understanding of sporulation and its regulation in TUAT1 would contribute to the development of a better inoculant using TUAT1.

### Data availability.

The complete genome sequence was deposited in GenBank under the accession number AP014928. The version described in this paper is the first version (AP014928.1). The sequences have been submitted to the Sequence Read Archive under the accession number PRJNA286715.
